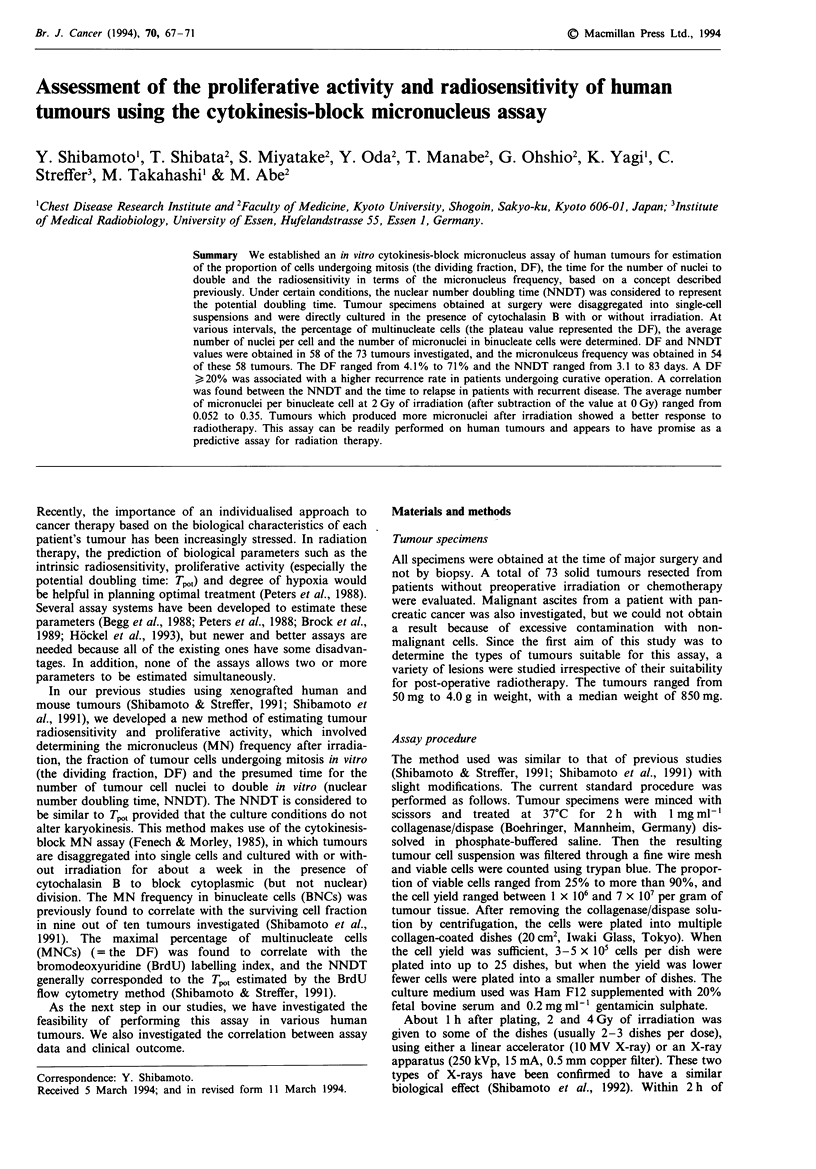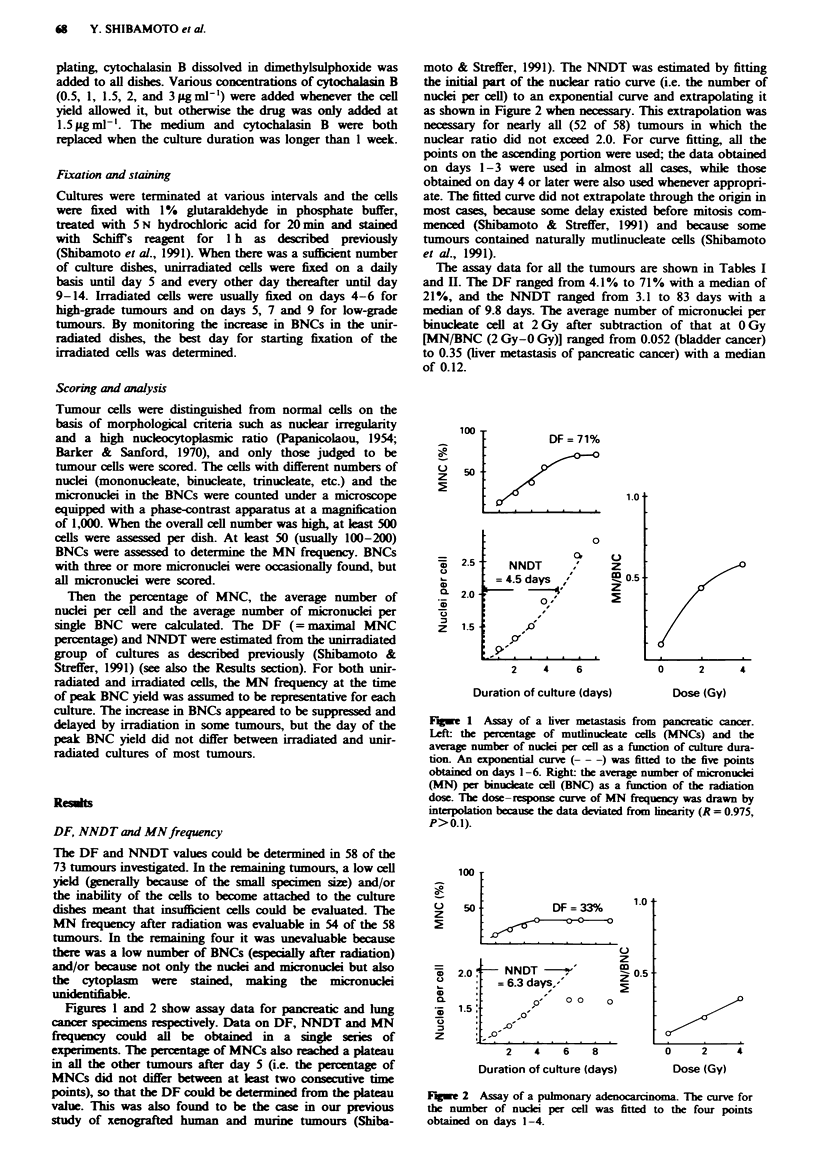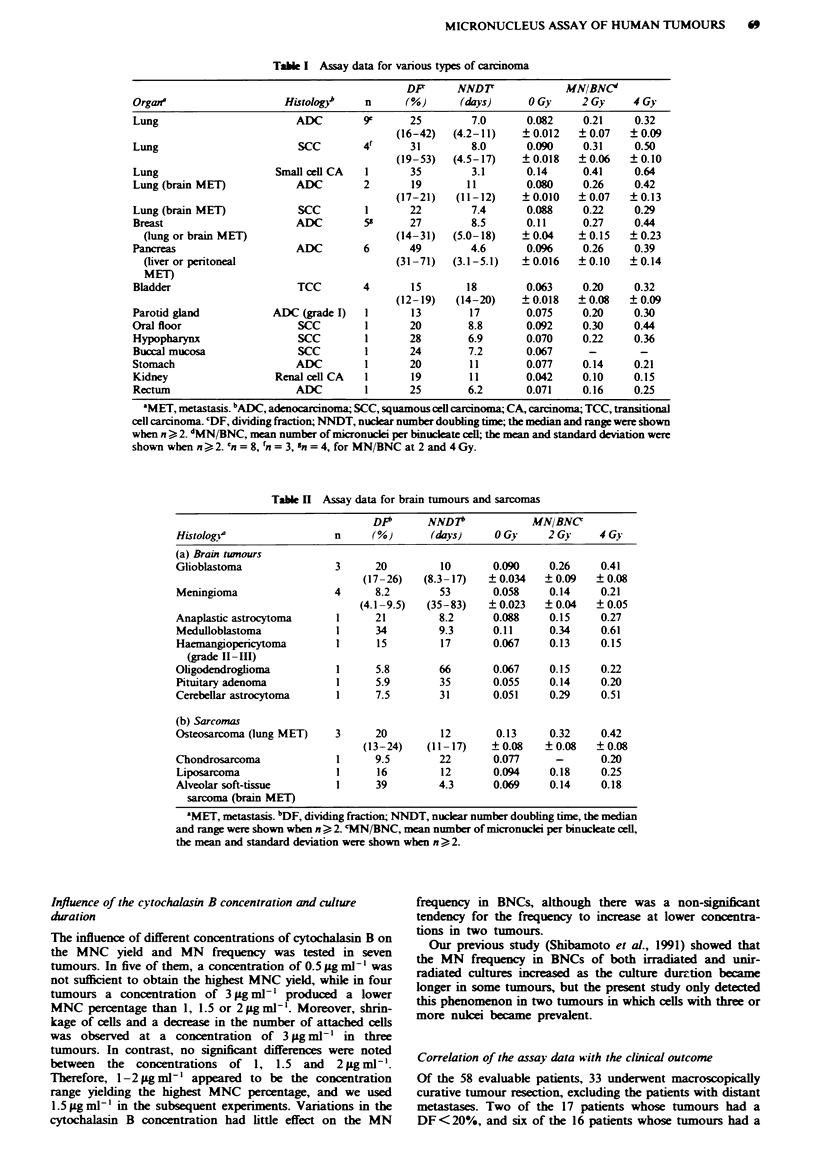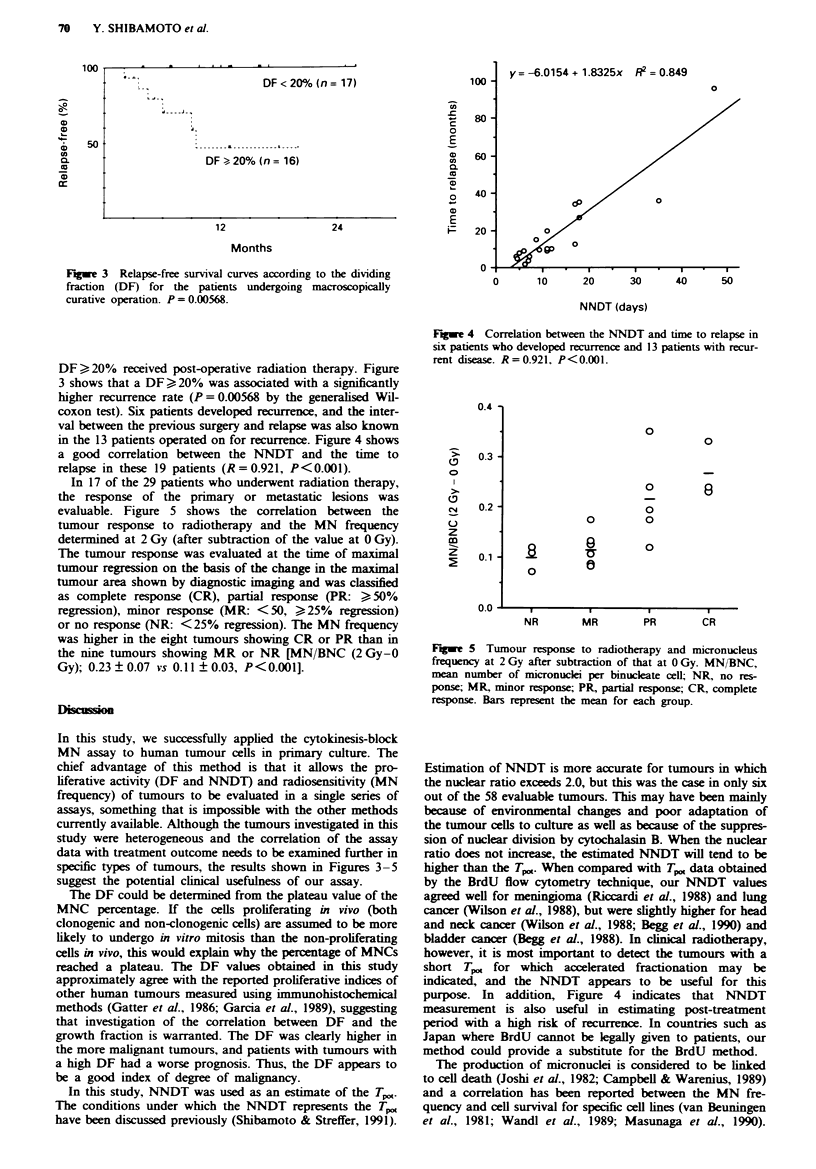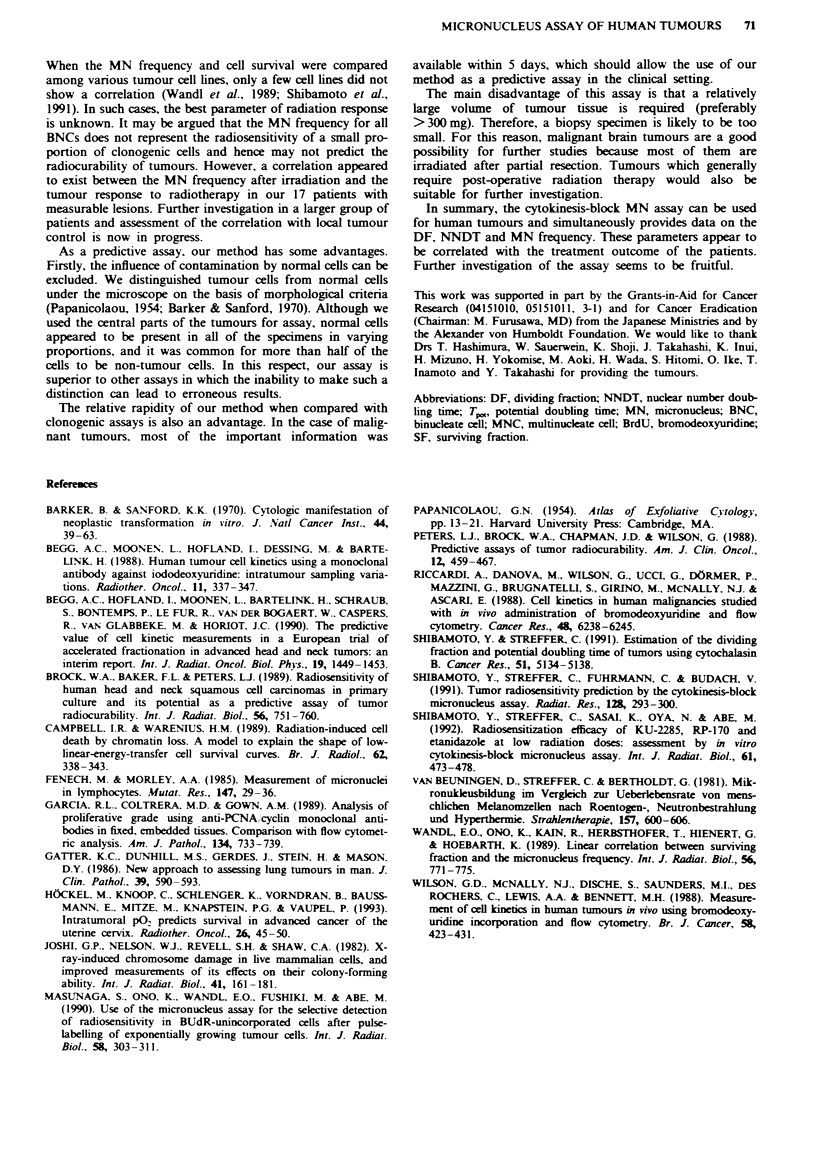# Assessment of the proliferative activity and radiosensitivity of human tumours using the cytokinesis-block micronucleus assay.

**DOI:** 10.1038/bjc.1994.251

**Published:** 1994-07

**Authors:** Y. Shibamoto, T. Shibata, S. Miyatake, Y. Oda, T. Manabe, G. Ohshio, K. Yagi, C. Streffer, M. Takahashi, M. Abe

**Affiliations:** Chest Disease Research Institute, Kyoto University, Japan.

## Abstract

We established an in vitro cytokinesis-block micronucleus assay of human tumours for estimation of the proportion of cells undergoing mitosis (the dividing fraction, DF), the time for the number of nuclei to double and the radiosensitivity in terms of the micronucleus frequency, based on a concept described previously. Under certain conditions, the nuclear number doubling time (NNDT) was considered to represent the potential doubling time. Tumour specimens obtained at surgery were disaggregated into single-cell suspensions and were directly cultured in the presence of cytochalasin B with or without irradiation. At various intervals, the percentage of multinucleate cells (the plateau value represented the DF), the average number of nuclei per cell and the number of micronuclei in binucleate cells were determined. DF and NNDT values were obtained in 58 of the 73 tumours investigated, and the micronucleus frequency was obtained in 54 of these 58 tumours. The DF ranged from 4.1% to 71% and the NNDT ranged from 3.1 to 83 days. A DF > or = 20% was associated with a higher recurrence rate in patients undergoing curative operation. A correlation was found between the NNDT and the time to relapse in patients with recurrent disease. The average number of micronuclei per binucleate cell at 2 Gy of irradiation (after subtraction of the value at 0 Gy) ranged from 0.052 to 0.35. Tumours which produced more micronuclei after irradiation showed a better response to radiotherapy. This assay can be readily performed on human tumours and appears to have promise as a predictive assay for radiation therapy.


Br. J. Cancer (1994), 70, 67–71 © Macmillan Press Ltd., 1994

### Assessment of the proliferative activity and radiosensitivity of human tumours using the cytokinesis-block micronucleus assay

Y. Shibamoto[1], T. Shibata[2], S. Miyatake[2], Y. Oda[2], T. Manabe[2], G. Ohshio[2], K. Yagi[1], C. Streffer[3], M. Takahashi[1] & M. Abe[2]

[1]*Chest Disease Research Institute and* [2]*Faculty of Medicine, Kyoto University, Shogoin, Sakyo-ku, Kyoto 606-01, Japan;* [3]*Institute of Medical Radiobiology, University of Essen, Hufelandstrasse 55, Essen 1, Germany.*

**Summary** We established an *in vitro* cytokinesis-block micronucleus assay of human tumours for estimation of the proportion of cells undergoing mitosis (the dividing fraction, DF), the time for the number of nuclei to double and the radiosensitivity in terms of the micronucleus frequency, based on a concept described previously. Under certain conditions, the nuclear number doubling time (NNDT) was considered to represent the potential doubling time. Tumour specimens obtained at surgery were disaggregated into single-cell suspensions and were directly cultured in the presence of cytochalasin B with or without irradiation. At various intervals, the percentage of multinucleate cells (the plateau value represented the DF), the average number of nuclei per cell and the number of micronuclei in binucleate cells were determined. DF and NNDT values were obtained in 58 of the 73 tumours investigated, and the micronulceus frequency was obtained in 54 of these 58 tumours. The DF ranged from 4.1% to 71% and the NNDT ranged from 3.1 to 83 days. A DF $\geqslant$20% was associated with a higher recurrence rate in patients undergoing curative operation. A correlation was found between the NNDT and the time to relapse in patients with recurrent disease. The average number of micronuclei per binucleate cell at 2 Gy of irradiation (after subtraction of the value at 0 Gy) ranged from 0.052 to 0.35. Tumours which produced more micronuclei after irradiation showed a better response to radiotherapy. This assay can be readily performed on human tumours and appears to have promise as a predictive assay for radiation therapy.

Recently, the importance of an individualised approach to cancer therapy based on the biological characteristics of each patient's tumour has been increasingly stressed. In radiation therapy, the prediction of biological parameters such as the intrinsic radiosensitivity, proliferative activity (especially the potential doubling time: $T_{pot}$) and degree of hypoxia would be helpful in planning optimal treatment (Peters *et al.*, 1988). Several assay systems have been developed to estimate these parameters (Begg *et al.*, 1988; Peters *et al.*, 1988; Brock *et al.*, 1989; Höckel *et al.*, 1993), but newer and better assays are needed because all of the existing ones have some disadvantages. In addition, none of the assays allows two or more parameters to be estimated simultaneously.

In our previous studies using xenografted human and mouse tumours (Shibamoto & Streffer, 1991; Shibamoto *et al.*, 1991), we developed a new method of estimating tumour radiosensitivity and proliferative activity, which involved determining the micronucleus (MN) frequency after irradiation, the fraction of tumour cells undergoing mitosis *in vitro* (the dividing fraction, DF) and the presumed time for the number of tumour cell nuclei to double *in vitro* (nuclear number doubling time, NNDT). The NNDT is considered to be similar to $T_{pot}$ provided that the culture conditions do not alter karyokinesis. This method makes use of the cytokinesis-block MN assay (Fenech & Morley, 1985), in which tumours are disaggregated into single cells and cultured with or without irradiation for about a week in the presence of cytochalasin B to block cytoplasmic (but not nuclear) division. The MN frequency in binucleate cells (BNCs) was previously found to correlate with the surviving cell fraction in nine out of ten tumours investigated (Shibamoto *et al.*, 1991). The maximal percentage of multinucleate cells (MNCs) (= the DF) was found to correlate with the bromodeoxyuridine (BrdU) labelling index, and the NNDT generally corresponded to the $T_{pot}$ estimated by the BrdU flow cytometry method (Shibamoto & Streffer, 1991).

As the next step in our studies, we have investigated the feasibility of performing this assay in various human tumours. We also investigated the correlation between assay data and clinical outcome.

Correspondence: Y. Shibamoto.
Received 5 March 1994; and in revised form 11 March 1994.

### Materials and methods

### *Tumour specimens*

All specimens were obtained at the time of major surgery and not by biopsy. A total of 73 solid tumours resected from patients without preoperative irradiation or chemotherapy were evaluated. Malignant ascites from a patient with pancreatic cancer was also investigated, but we could not obtain a result because of excessive contamination with non-malignant cells. Since the first aim of this study was to determine the types of tumours suitable for this assay, a variety of lesions were studied irrespective of their suitability for post-operative radiotherapy. The tumours ranged from 50 mg to 4.0 g in weight, with a median weight of 850 mg.

### *Assay procedure*

The method used was similar to that of previous studies (Shibamoto & Streffer, 1991; Shibamoto *et al.*, 1991) with slight modifications. The current standard procedure was performed as follows. Tumour specimens were minced with scissors and treated at 37°C for 2 h with 1 mg ml$^{-1}$ collagenase/dispase (Boehringer, Mannheim, Germany) dissolved in phosphate-buffered saline. Then the resulting tumour cell suspension was filtered through a fine wire mesh and viable cells were counted using trypan blue. The proportion of viable cells ranged from 25% to more than 90%, and the cell yield ranged between $1 \times 10^6$ and $7 \times 10^7$ per gram of tumour tissue. After removing the collagenase/dispase solution by centrifugation, the cells were plated into multiple collagen-coated dishes (20 cm$^2$, Iwaki Glass, Tokyo). When the cell yield was sufficient, $3-5 \times 10^5$ cells per dish were plated into up to 25 dishes, but when the yield was lower fewer cells were plated into a smaller number of dishes. The culture medium used was Ham F12 supplemented with 20% fetal bovine serum and 0.2 mg ml$^{-1}$ gentamicin sulphate.

About 1 h after plating, 2 and 4 Gy of irradiation was given to some of the dishes (usually 2–3 dishes per dose), using either a linear accelerator (10 MV X-ray) or an X-ray apparatus (250 kVp, 15 mA, 0.5 mm copper filter). These two types of X-rays have been confirmed to have a similar biological effect (Shibamoto *et al.*, 1992). Within 2 h of plating, cytochalasin B dissolved in dimethylsulphoxide was added to all dishes. Various concentrations of cytochalasin B (0.5, 1, 1.5, 2, and 3 $\mu g\ ml^{-1}$) were added whenever the cell yield allowed it, but otherwise the drug was only added at 1.5 $\mu g\ ml^{-1}$. The medium and cytochalasin B were both replaced when the culture duration was longer than 1 week.

*Fixation and staining*

Cultures were terminated at various intervals and the cells were fixed with 1% glutaraldehyde in phosphate buffer, treated with 5 N hydrochloric acid for 20 min and stained with Schiff's reagent for 1 h as described previously (Shibamoto *et al.*, 1991). When there was a sufficient number of culture dishes, unirradiated cells were fixed on a daily basis until day 5 and every other day thereafter until day 9-14. Irradiated cells were usually fixed on days 4-6 for high-grade tumours and on days 5, 7 and 9 for low-grade tumours. By monitoring the increase in BNCs in the unirradiated dishes, the best day for starting fixation of the irradiated cells was determined.

*Scoring and analysis*

Tumour cells were distinguished from normal cells on the basis of morphological criteria such as nuclear irregularity and a high nucleocytoplasmic ratio (Papanicolaou, 1954; Barker & Sanford, 1970), and only those judged to be tumour cells were scored. The cells with different numbers of nuclei (mononucleate, binucleate, trinucleate, etc.) and the micronuclei in the BNCs were counted under a microscope equipped with a phase-contrast apparatus at a magnification of 1,000. When the overall cell number was high, at least 500 cells were assessed per dish. At least 50 (usually 100-200) BNCs were assessed to determine the MN frequency. BNCs with three or more micronuclei were occasionally found, but all micronuclei were scored.

Then the percentage of MNC, the average number of nuclei per cell and the average number of micronuclei per single BNC were calculated. The DF (= maximal MNC percentage) and NNDT were estimated from the unirradiated group of cultures as described previously (Shibamoto & Streffer, 1991) (see also the Results section). For both unirradiated and irradiated cells, the MN frequency at the time of peak BNC yield was assumed to be representative for each culture. The increase in BNCs appeared to be suppressed and delayed by irradiation in some tumours, but the day of the peak BNC yield did not differ between irradiated and unirradiated cultures of most tumours.

### Results

*DF, NNDT and MN frequency*

The DF and NNDT values could be determined in 58 of the 73 tumours investigated. In the remaining tumours, a low cell yield (generally because of the small specimen size) and/or the inability of the cells to become attached to the culture dishes meant that insufficient cells could be evaluated. The MN frequency after radiation was evaluable in 54 of the 58 tumours. In the remaining four it was unevaluable because there was a low number of BNCs (especially after radiation) and/or because not only the nuclei and micronuclei but also the cytoplasm were stained, making the micronuclei unidentifiable.

Figures 1 and 2 show assay data for pancreatic and lung cancer specimens respectively. Data on DF, NNDT and MN frequency could all be obtained in a single series of experiments. The percentage of MNCs also reached a plateau in all the other tumours after day 5 (i.e. the percentage of MNCs did not differ between at least two consecutive time points), so that the DF could be determined from the plateau value. This was also found to be the case in our previous study of xenografted human and murine tumours (Shibamoto & Streffer, 1991). The NNDT was estimated by fitting the initial part of the nuclear ratio curve (i.e. the number of nuclei per cell) to an exponential curve and extrapolating it as shown in Figure 2 when necessary. This extrapolation was necessary for nearly all (52 of 58) tumours in which the nuclear ratio did not exceed 2.0. For curve fitting, all the points on the ascending portion were used; the data obtained on days 1-3 were used in almost all cases, while those obtained on day 4 or later were also used whenever appropriate. The fitted curve did not extrapolate through the origin in most cases, because some delay existed before mitosis commenced (Shibamoto & Streffer, 1991) and because some tumours contained naturally mutlinucleate cells (Shibamoto *et al.*, 1991).

The assay data for all the tumours are shown in Tables I and II. The DF ranged from 4.1% to 71% with a median of 21%, and the NNDT ranged from 3.1 to 83 days with a median of 9.8 days. The average number of micronuclei per binucleate cell at 2 Gy after subtraction of that at 0 Gy [MN/BNC (2 Gy-0 Gy)] ranged from 0.052 (bladder cancer) to 0.35 (liver metastasis of pancreatic cancer) with a median of 0.12.

**Figure 1** Assay of a liver metastasis from pancreatic cancer. Left: the percentage of mutlinucleate cells (MNCs) and the average number of nuclei per cell as a function of culture duration. An exponential curve (- - -) was fitted to the five points obtained on days 1-6. Right: the average number of micronuclei (MN) per binucleate cell (BNC) as a function of the radiation dose. The dose-response curve of MN frequency was drawn by interpolation because the data deviated from linearity ($R = 0.975$, $P > 0.1$).

**Figure 2** Assay of a pulmonary adenocarcinoma. The curve for the number of nuclei per cell was fitted to the four points obtained on days 1-4.

**Table I** Assay data for various types of carcinoma

| *Organ*[a] | *Histology*[b] | n | *DF*[c] (%) | *NNDT*[c] (days) | *MN/BNC*[d] 0 Gy | 2 Gy | 4 Gy |
|---|---|---|---|---|---|---|---|
| Lung | ADC | 9[e] | 25 | 7.0 | 0.082 | 0.21 | 0.32 |
| | | | (16–42) | (4.2–11) | ± 0.012 | ± 0.07 | ± 0.09 |
| Lung | SCC | 4[f] | 31 | 8.0 | 0.090 | 0.31 | 0.50 |
| | | | (19–53) | (4.5–17) | ± 0.018 | ± 0.06 | ± 0.10 |
| Lung | Small cell CA | 1 | 35 | 3.1 | 0.14 | 0.41 | 0.64 |
| Lung (brain MET) | ADC | 2 | 19 | 11 | 0.080 | 0.26 | 0.42 |
| | | | (17–21) | (11–12) | ± 0.010 | ± 0.07 | ± 0.13 |
| Lung (brain MET) | SCC | 1 | 22 | 7.4 | 0.088 | 0.22 | 0.29 |
| Breast | ADC | 5[g] | 27 | 8.5 | 0.11 | 0.27 | 0.44 |
| (lung or brain MET) | | | (14–31) | (5.0–18) | ± 0.04 | ± 0.15 | ± 0.23 |
| Pancreas | ADC | 6 | 49 | 4.6 | 0.096 | 0.26 | 0.39 |
| (liver or peritoneal MET) | | | (31–71) | (3.1–5.1) | ± 0.016 | ± 0.10 | ± 0.14 |
| Bladder | TCC | 4 | 15 | 18 | 0.063 | 0.20 | 0.32 |
| | | | (12–19) | (14–20) | ± 0.018 | ± 0.08 | ± 0.09 |
| Parotid gland | ADC (grade I) | 1 | 13 | 17 | 0.075 | 0.20 | 0.30 |
| Oral floor | SCC | 1 | 20 | 8.8 | 0.092 | 0.30 | 0.44 |
| Hypopharynx | SCC | 1 | 28 | 6.9 | 0.070 | 0.22 | 0.36 |
| Buccal mucosa | SCC | 1 | 24 | 7.2 | 0.067 | – | – |
| Stomach | ADC | 1 | 20 | 11 | 0.077 | 0.14 | 0.21 |
| Kidney | Renal cell CA | 1 | 19 | 11 | 0.042 | 0.10 | 0.15 |
| Rectum | ADC | 1 | 25 | 6.2 | 0.071 | 0.16 | 0.25 |

[a]MET, metastasis. [b]ADC, adenocarcinoma; SCC, squamous cell carcinoma; CA, carcinoma; TCC, transitional cell carcinoma. [c]DF, dividing fraction; NNDT, nuclear number doubling time; the median and range were shown when $n \geqslant 2$. [d]MN/BNC, mean number of micronuclei per binucleate cell; the mean and standard deviation were shown when $n \geqslant 2$. [e]$n = 8$, [f]$n = 3$, [g]$n = 4$, for MN/BNC at 2 and 4 Gy.

**Table II** Assay data for brain tumours and sarcomas

| *Histology*[a] | n | *DF*[b] (%) | *NNDT*[b] (days) | *MN/BNC*[c] 0 Gy | 2 Gy | 4 Gy |
|---|---|---|---|---|---|---|
| (a) *Brain tumours* | | | | | | |
| Glioblastoma | 3 | 20 | 10 | 0.090 | 0.26 | 0.41 |
| | | (17–26) | (8.3–17) | ± 0.034 | ± 0.09 | ± 0.08 |
| Meningioma | 4 | 8.2 | 53 | 0.058 | 0.14 | 0.21 |
| | | (4.1–9.5) | (35–83) | ± 0.023 | ± 0.04 | ± 0.05 |
| Anaplastic astrocytoma | 1 | 21 | 8.2 | 0.088 | 0.15 | 0.27 |
| Medulloblastoma | 1 | 34 | 9.3 | 0.11 | 0.34 | 0.61 |
| Haemangiopericytoma (grade II–III) | 1 | 15 | 17 | 0.067 | 0.13 | 0.15 |
| Oligodendroglioma | 1 | 5.8 | 66 | 0.067 | 0.15 | 0.22 |
| Pituitary adenoma | 1 | 5.9 | 35 | 0.055 | 0.14 | 0.20 |
| Cerebellar astrocytoma | 1 | 7.5 | 31 | 0.051 | 0.29 | 0.51 |
| (b) *Sarcomas* | | | | | | |
| Osteosarcoma (lung MET) | 3 | 20 | 12 | 0.13 | 0.32 | 0.42 |
| | | (13–24) | (11–17) | ± 0.08 | ± 0.08 | ± 0.08 |
| Chondrosarcoma | 1 | 9.5 | 22 | 0.077 | – | 0.20 |
| Liposarcoma | 1 | 16 | 12 | 0.094 | 0.18 | 0.25 |
| Alveolar soft-tissue sarcoma (brain MET) | 1 | 39 | 4.3 | 0.069 | 0.14 | 0.18 |

[a]MET, metastasis. [b]DF, dividing fraction; NNDT, nuclear number doubling time, the median and range were shown when $n \geqslant 2$. [c]MN/BNC, mean number of micronuclei per binucleate cell, the mean and standard deviation were shown when $n \geqslant 2$.

### *Influence of the cytochalasin B concentration and culture duration*

The influence of different concentrations of cytochalasin B on the MNC yield and MN frequency was tested in seven tumours. In five of them, a concentration of 0.5 μg ml$^{-1}$ was not sufficient to obtain the highest MNC yield, while in four tumours a concentration of 3 μg ml$^{-1}$ produced a lower MNC percentage than 1, 1.5 or 2 μg ml$^{-1}$. Moreover, shrinkage of cells and a decrease in the number of attached cells was observed at a concentration of 3 μg ml$^{-1}$ in three tumours. In contrast, no significant differences were noted between the concentrations of 1, 1.5 and 2 μg ml$^{-1}$. Therefore, 1–2 μg ml$^{-1}$ appeared to be the concentration range yielding the highest MNC percentage, and we used 1.5 μg ml$^{-1}$ in the subsequent experiments. Variations in the cytochalasin B concentration had little effect on the MN frequency in BNCs, although there was a non-significant tendency for the frequency to increase at lower concentrations in two tumours.

Our previous study (Shibamoto *et al.*, 1991) showed that the MN frequency in BNCs of both irradiated and unirradiated cultures increased as the culture duration became longer in some tumours, but the present study only detected this phenomenon in two tumours in which cells with three or more nulcei became prevalent.

### *Correlation of the assay data with the clinical outcome*

Of the 58 evaluable patients, 33 underwent macroscopically curative tumour resection, excluding the patients with distant metastases. Two of the 17 patients whose tumours had a DF<20%, and six of the 16 patients whose tumours had a

**Figure 3** Relapse-free survival curves according to the dividing fraction (DF) for the patients undergoing macroscopically curative operation. $P = 0.00568$.

**Figure 4** Correlation between the NNDT and time to relapse in six patients who developed recurrence and 13 patients with recurrent disease. $R = 0.921$, $P < 0.001$.

**Figure 5** Tumour response to radiotherapy and micronucleus frequency at 2 Gy after subtraction of that at 0 Gy. MN/BNC, mean number of micronuclei per binucleate cell; NR, no response; MR, minor response; PR, partial response; CR, complete response. Bars represent the mean for each group.

DF ≥ 20% received post-operative radiation therapy. Figure 3 shows that a DF ≥ 20% was associated with a significantly higher recurrence rate ($P = 0.00568$ by the generalised Wilcoxon test). Six patients developed recurrence, and the interval between the previous surgery and relapse was also known in the 13 patients operated on for recurrence. Figure 4 shows a good correlation between the NNDT and the time to relapse in these 19 patients ($R = 0.921$, $P < 0.001$).

In 17 of the 29 patients who underwent radiation therapy, the response of the primary or metastatic lesions was evaluable. Figure 5 shows the correlation between the tumour response to radiotherapy and the MN frequency determined at 2 Gy (after subtraction of the value at 0 Gy). The tumour response was evaluated at the time of maximal tumour regression on the basis of the change in the maximal tumour area shown by diagnostic imaging and was classified as complete response (CR), partial response (PR: ≥50% regression), minor response (MR: <50, ≥25% regression) or no response (NR: <25% regression). The MN frequency was higher in the eight tumours showing CR or PR than in the nine tumours showing MR or NR [MN/BNC (2 Gy−0 Gy); $0.23 \pm 0.07$ vs $0.11 \pm 0.03$, $P < 0.001$].

### Discussion

In this study, we successfully applied the cytokinesis-block MN assay to human tumour cells in primary culture. The chief advantage of this method is that it allows the proliferative activity (DF and NNDT) and radiosensitivity (MN frequency) of tumours to be evaluated in a single series of assays, something that is impossible with the other methods currently available. Although the tumours investigated in this study were heterogeneous and the correlation of the assay data with treatment outcome needs to be examined further in specific types of tumours, the results shown in Figures 3–5 suggest the potential clinical usefulness of our assay.

The DF could be determined from the plateau value of the MNC percentage. If the cells proliferating *in vivo* (both clonogenic and non-clonogenic cells) are assumed to be more likely to undergo *in vitro* mitosis than the non-proliferating cells *in vivo*, this would explain why the percentage of MNCs reached a plateau. The DF values obtained in this study approximately agree with the reported proliferative indices of other human tumours measured using immunohistochemical methods (Gatter *et al.*, 1986; Garcia *et al.*, 1989), suggesting that investigation of the correlation between DF and the growth fraction is warranted. The DF was clearly higher in the more malignant tumours, and patients with tumours with a high DF had a worse prognosis. Thus, the DF appears to be a good index of degree of malignancy.

In this study, NNDT was used as an estimate of the $T_{pot}$. The conditions under which the NNDT represents the $T_{pot}$ have been discussed previously (Shibamoto & Streffer, 1991). Estimation of NNDT is more accurate for tumours in which the nuclear ratio exceeds 2.0, but this was the case in only six out of the 58 evaluable tumours. This may have been mainly because of environmental changes and poor adaptation of the tumour cells to culture as well as because of the suppression of nuclear division by cytochalasin B. When the nuclear ratio does not increase, the estimated NNDT will tend to be higher than the $T_{pot}$. When compared with $T_{pot}$ data obtained by the BrdU flow cytometry technique, our NNDT values agreed well for meningioma (Riccardi *et al.*, 1988) and lung cancer (Wilson *et al.*, 1988), but were slightly higher for head and neck cancer (Wilson *et al.*, 1988; Begg *et al.*, 1990) and bladder cancer (Begg *et al.*, 1988). In clinical radiotherapy, however, it is most important to detect the tumours with a short $T_{pot}$ for which accelerated fractionation may be indicated, and the NNDT appears to be useful for this purpose. In addition, Figure 4 indicates that NNDT measurement is also useful in estimating post-treatment period with a high risk of recurrence. In countries such as Japan where BrdU cannot be legally given to patients, our method could provide a substitute for the BrdU method.

The production of micronuclei is considered to be linked to cell death (Joshi *et al.*, 1982; Campbell & Warenius, 1989) and a correlation has been reported between the MN frequency and cell survival for specific cell lines (van Beuningen *et al.*, 1981; Wandl *et al.*, 1989; Masunaga *et al.*, 1990).

When the MN frequency and cell survival were compared among various tumour cell lines, only a few cell lines did not show a correlation (Wandl *et al.*, 1989; Shibamoto *et al.*, 1991). In such cases, the best parameter of radiation response is unknown. It may be argued that the MN frequency for all BNCs does not represent the radiosensitivity of a small proportion of clonogenic cells and hence may not predict the radiocurability of tumours. However, a correlation appeared to exist between the MN frequency after irradiation and the tumour response to radiotherapy in our 17 patients with measurable lesions. Further investigation in a larger group of patients and assessment of the correlation with local tumour control is now in progress.

As a predictive assay, our method has some advantages. Firstly, the influence of contamination by normal cells can be excluded. We distinguished tumour cells from normal cells under the microscope on the basis of morphological criteria (Papanicolaou, 1954; Barker & Sanford, 1970). Although we used the central parts of the tumours for assay, normal cells appeared to be present in all of the specimens in varying proportions, and it was common for more than half of the cells to be non-tumour cells. In this respect, our assay is superior to other assays in which the inability to make such a distinction can lead to erroneous results.

The relative rapidity of our method when compared with clonogenic assays is also an advantage. In the case of malignant tumours, most of the important information was available within 5 days, which should allow the use of our method as a predictive assay in the clinical setting.

The main disadvantage of this assay is that a relatively large volume of tumour tissue is required (preferably $>300$ mg). Therefore, a biopsy specimen is likely to be too small. For this reason, malignant brain tumours are a good possibility for further studies because most of them are irradiated after partial resection. Tumours which generally require post-operative radiation therapy would also be suitable for further investigation.

In summary, the cytokinesis-block MN assay can be used for human tumours and simultaneously provides data on the DF, NNDT and MN frequency. These parameters appear to be correlated with the treatment outcome of the patients. Further investigation of the assay seems to be fruitful.

This work was supported in part by the Grants-in-Aid for Cancer Research (04151010, 05151011, 3-1) and for Cancer Eradication (Chairman: M. Furusawa, MD) from the Japanese Ministries and by the Alexander von Humboldt Foundation. We would like to thank Drs T. Hashimura, W. Sauerwein, K. Shoji, J. Takahashi, K. Inui, H. Mizuno, H. Yokomise, M. Aoki, H. Wada, S. Hitomi, O. Ike, T. Inamoto and Y. Takahashi for providing the tumours.

Abbreviations: DF, dividing fraction; NNDT, nuclear number doubling time; $T_{pot}$, potential doubling time; MN, micronucleus; BNC, binucleate cell; MNC, multinucleate cell; BrdU, bromodeoxyuridine; SF, surviving fraction.

**References**

BARKER, B. & SANFORD, K.K. (1970). Cytologic manifestation of neoplastic transformation *in vitro*. *J. Natl Cancer Inst.*, **44**, 39-63.

BEGG, A.C., MOONEN, L., HOFLAND, I., DESSING, M. & BARTELINK, H. (1988). Human tumour cell kinetics using a monoclonal antibody against iododeoxyuridine: intratumour sampling variations. *Radiother. Oncol.*, **11**, 337-347.

BEGG, A.C., HOFLAND, I., MOONEN, L., BARTELINK, H., SCHRAUB, S., BONTEMPS, P., LE FUR, R., VAN DER BOGAERT, W., CASPERS, R., VAN GLABBEKE, M. & HORIOT, J.C. (1990). The predictive value of cell kinetic measurements in a European trial of accelerated fractionation in advanced head and neck tumors: an interim report. *Int. J. Radiat. Oncol. Biol. Phys.*, **19**, 1449-1453.

BROCK, W.A., BAKER, F.L. & PETERS, L.J. (1989). Radiosensitivity of human head and neck squamous cell carcinomas in primary culture and its potential as a predictive assay of tumor radiocurability. *Int. J. Radiat. Biol.*, **56**, 751-760.

CAMPBELL, I.R. & WARENIUS, H.M. (1989). Radiation-induced cell death by chromatin loss. A model to explain the shape of low-linear-energy-transfer cell survival curves. *Br. J. Radiol.*, **62**, 338-343.

FENECH, M. & MORLEY, A.A. (1985). Measurement of micronuclei in lymphocytes. *Mutat. Res.*, **147**, 29-36.

GARCIA, R.L., COLTRERA, M.D. & GOWN, A.M. (1989). Analysis of proliferative grade using anti-PCNA/cyclin monoclonal antibodies in fixed, embedded tissues. Comparison with flow cytometric analysis. *Am. J. Pathol.*, **134**, 733-739.

GATTER, K.C., DUNHILL, M.S., GERDES, J., STEIN, H. & MASON, D.Y. (1986). New approach to assessing lung tumours in man. *J. Clin. Pathol.*, **39**, 590-593.

HÖCKEL, M., KNOOP, C., SCHLENGER, K., VORNDRAN, B., BAUSSMANN, E., MITZE, M., KNAPSTEIN, P.G. & VAUPEL, P. (1993). Intratumoral $pO_2$ predicts survival in advanced cancer of the uterine cervix. *Radiother. Oncol.*, **26**, 45-50.

JOSHI, G.P., NELSON, W.J., REVELL, S.H. & SHAW, C.A. (1982). X-ray-induced chromosome damage in live mammalian cells, and improved measurements of its effects on their colony-forming ability. *Int. J. Radiat. Biol.*, **41**, 161-181.

MASUNAGA, S., ONO, K., WANDL, E.O., FUSHIKI, M. & ABE, M. (1990). Use of the micronucleus assay for the selective detection of radiosensitivity in BUdR-unincorporated cells after pulse-labelling of exponentially growing tumour cells. *Int. J. Radiat. Biol.*, **58**, 303-311.

PAPANICOLAOU, G.N. (1954). *Atlas of Exfoliative Cytology*, pp. 13-21. Harvard University Press: Cambridge, MA.

PETERS, L.J., BROCK, W.A., CHAPMAN, J.D. & WILSON, G. (1988). Predictive assays of tumor radiocurability. *Am. J. Clin. Oncol.*, **12**, 459-467.

RICCARDI, A., DANOVA, M., WILSON, G., UCCI, G., DÖRMER, P., MAZZINI, G., BRUGNATELLI, S., GIRINO, M., MCNALLY, N.J. & ASCARI, E. (1988). Cell kinetics in human malignancies studied with *in vivo* administration of bromodeoxyuridine and flow cytometry. *Cancer Res.*, **48**, 6238-6245.

SHIBAMOTO, Y. & STREFFER, C. (1991). Estimation of the dividing fraction and potential doubling time of tumors using cytochalasin B. *Cancer Res.*, **51**, 5134-5138.

SHIBAMOTO, Y., STREFFER, C., FUHRMANN, C. & BUDACH, V. (1991). Tumor radiosensitivity prediction by the cytokinesis-block micronucleus assay. *Radiat. Res.*, **128**, 293-300.

SHIBAMOTO, Y., STREFFER, C., SASAI, K., OYA, N. & ABE, M. (1992). Radiosensitization efficacy of KU-2285, RP-170 and etanidazole at low radiation doses: assessment by *in vitro* cytokinesis-block micronucleus assay. *Int. J. Radiat. Biol.*, **61**, 473-478.

VAN BEUNINGEN, D., STREFFER, C. & BERTHOLDT, G. (1981). Mikronukleusbildung im Vergleich zur Ueberlebensrate von menschlichen Melanomzellen nach Roentogen-, Neutronbestrahlung und Hyperthermie. *Strahlentherapie*, **157**, 600-606.

WANDL, E.O., ONO, K., KAIN, R., HERBSTHOFER, T., HIENERT, G. & HOEBARTH, K. (1989). Linear correlation between surviving fraction and the micronucleus frequency. *Int. J. Radiat. Biol.*, **56**, 771-775.

WILSON, G.D., MCNALLY, N.J., DISCHE, S., SAUNDERS, M.I., DES ROCHERS, C., LEWIS, A.A. & BENNETT, M.H. (1988). Measurement of cell kinetics in human tumours *in vivo* using bromodeoxyuridine incorporation and flow cytometry. *Br. J. Cancer*, **58**, 423-431.